# Prediction of out-of-hospital cardiac arrest in older patients with insomnia: a longitudinal population study

**DOI:** 10.1186/s12877-025-06285-x

**Published:** 2025-08-07

**Authors:** Chih-Wei Sung, Cheng-Che Chen, Yun-Ting Chih, Cheng-Yi Fan, Edward Pei-Chuan Huang

**Affiliations:** 1 Department of Emergency Medicine, National Taiwan University Hospital Hsin-Chu Branch, Hsinchu, Taiwan; 2 Department of Emergency Medicine, College of Medicine, National Taiwan University, Taipei, Taiwan; 3 Department of Psychiatry, National Taiwan University Hospital Hsin-Chu Branch, Hsinchu, Taiwan; 4 Department of Psychiatry, College of Medicine, National Taiwan University, Taipei, Taiwan; 5 Institute of Molecular Medicine, National Tsing Hua University, Hsinchu, Taiwan; 6 Department of Emergency Medicine, National Taiwan University Hospital, Taipei, Taiwan

**Keywords:** Out-of-hospital cardiac arrest, Prediction model, Insomnia, Older patients, National health insurance research database

## Abstract

**Background:**

The association of insomnia in older patients with out-of-hospital cardiac arrest (OHCA) is not completely elucidated. The current study developed and validated a predictive model for OHCA in older patients using population-based analysis.

**Methods:**

This study used data from the National Health Insurance research database. The cohort included older patients (aged more than 65 years) diagnosed with insomnia and treated with insomnia medications. The multivariate logistic regression model was used to analyze potential OHCA predictors. The model’s performance was evaluated via internal and external validations using the receiver operating characteristic curve and confusion matrix indices.

**Results:**

Of the 438,147 older patients with insomnia, 6,931 (1.6%) experienced OHCA. The key predictors included age, male sex, previous use of medical resources, treatment with hemodialysis, existing comorbidities, medication possession ratio, medication changes, and recent psychotherapy. The receiver operating characteristic curve values of the predictive models for 7-, 30-, and 90-day OHCA ranged from 0.757 to 0.787. The 2019 and 2020 external validation confirmed that the model was robust. The sensitivity, specificity, positive likelihood ratio, negative likelihood ratio, and diagnostic odds ratio of the 7-day model in 2019 were 0.781, 0.754, 2.78, 0.42, and 6.58, respectively. Meanwhile, the sensitivity, specificity, positive likelihood ratio, negative likelihood ratio, and diagnostic odds ratio of the 7-day model in 2020 were 0.731, 0.677, 2.27, 0.40, and 5.71, respectively.

**Conclusions:**

This study developed a robust predictive model for OHCA among older patients with insomnia. The model was effective in identifying important predictors that could assist psychiatrists in recognizing high-risk individuals and enhancing preventive care.

**Supplementary Information:**

The online version contains supplementary material available at 10.1186/s12877-025-06285-x.

## Introduction

Insomnia is the primary sleep disorder observed among older individuals visiting geriatric clinics [1]. The prevalence of insomnia symptoms in this population is approximately 30–48% [2]. Geriatric insomnia can affect quality of life. Further, it is associated with various comorbidities that can lead to sudden cardiac-related death, a specific type of out-of-hospital cardiac arrest (OHCA).

Previous research has shown that insomnia is associated with increased mortality from conditions such as cancer [3]suicidal tendencies [4]hypertension, and myocardial infarction [5, 6]. However, its association with OHCA, particularly in the geriatric clinic population, is less documented. Miner et al. revealed that sleep disruption, a specific type of sleep disorder, is associated with increased ventricular ectopy and a higher frequency of cardiac arrest [7]. Meanwhile, a meta-analysis adjusted for age and other cardiovascular risk factors presented the risk ratios for heart disease caused by insomnia symptoms [8]. Further, Jung et al. investigated the association between insomnia and the incidence of OHCA, and results showed no significant association between them. However, the study had several significant limitations. It had a small sample size, so selection bias might have existed in the cohort [9]. The notion that early and proactive medical intervention for insomnia can reduce OHCA risk remains a subject of debate.

The association between insomnia in older patients and OHCA is not completely explored in the existing literature. The impact of various factors such as existing comorbidities, adherence to insomnia medication regimens, medication modifications, and the role of mental health counseling on the incidence of OHCA remains controversial. OHCA in the older population is challenging to predict. This is because OHCA has a low incidence rate. Hence, further studies require large sample sizes. Moreover, there is a multifactorial complexity involving daily health fluctuations and multiple concurrent comorbidities [10]. To address these issues, this research conducted a longitudinal population analysis to develop a robust predictive model for both short- and long-term OHCA events and their probability among older patients with insomnia. This model, which is customized for Asian populations, incorporates temporal validation to enhance its accuracy and reliability.

## Methods

### Study setting and data source

This retrospective population-based cohort study utilized a dynamic repeated measurement approach. The initial observation date was established 90 days after the enrollment start date, with subsequent observations occurring every 30 days until the conclusion of the study period. The enrollment start date was defined as the date of the first outpatient visit involving the prescription of insomnia medications, starting from January 1, 2011. Each observation date was as an independent point for assessing OHCA probability.

The data were collected from the Taiwan’s Health and Welfare Data Science Center (HWDC), supervised by the Ministry of Health and Welfare. The HWDC provides anonymized data, secured using stringent protocols including monitored access, thereby eliminating the need for patient consent. The data from the National Health Insurance (NHI) research database within the HWDC were examined. The Taiwan’s NHI system, which was established in 1995, covered 99% of the population by 2004 [11]. This database contains detailed demographic and clinical data of all participants, coded according to the International Classification of Diseases, 9th (ICD-9) or 10th (ICD-10) Edition. It includes extensive records of healthcare utilization, medical expenditures, and socioeconomic information for all enrollees [12–14].

### Identification of insomnia in older patients and inclusion and exclusion criteria

In the Taiwan’s NHI system, outpatient visits typically involve medical prescriptions that last for at least 7 days for a specific illness. From January 2013 to December 2020, cases of insomnia were identified by integrating diagnostic codes with medication prescriptions. Insomnia was identified using the ICD-9 codes 307.41, 307.42, 780.51, and 780.52 and the ICD-10 codes G47.0 and F51.0. The NHI’s auditing system, which checks for fraudulent activities and ensures the reliability of the medical care data, was also utilized.

To determine the need for insomnia medications, the number of outpatient visits per patient in the previous year that involved a prescription for at least 7 days, according to the NHI standards, was evaluated. A participant who had more than two visits was considered to have a need for insomnia medications in any given year.

The inclusion criteria were as follows: (1) older adults, defined a priori as individuals aged 65 years or older, (2) those with insomnia diagnosed using the diagnostic codes, and (3) those with prescription for insomnia medications. Only older patients with documented insomnia diagnostic codes were further examined for their medication use. A double-check mechanism was implemented to ensure the accuracy of the study population for analysis. There were 44 drugs prescribed for insomnia (Supplementary Table 1), and these drugs were from five classifications (benzodiazepines, sedatives, antipsychotics, antidepressants, and miscellaneous). The exclusion criteria were as follows: (1) patients who started treatment after December 24, 2020, and (2) those who died within 7 days after their first entry.

### Definition of predictors

This study assessed the potential predictors of OHCA in a older population with insomnia, focusing on variables such as age, sex, pre-existing comorbidities, presence of urgent medical needs, hemodialysis status, medication possession ratio (MPR), medication changes, psychotherapy, and the month of observation. All predictors, including comorbidities retained in the final models, were identified a priori based on (i) established pathophysiological links to OHCA reported in the literature, (ii) recommendations from three board-certified emergency physicians and a senior psychiatrist specializing in geriatric medicine, and (iii) findings from our nationwide epidemiological study of OHCA risk factors in Taiwan [15].

Pre-existing comorbidities were identified at the start of each year by analyzing comprehensive prescription data from the NHI system, which included conditions such as diabetes mellitus, hypertension, chronic kidney disease, peptic ulcer disease, heart disease, cerebrovascular disease, hyperlipidemia, chronic obstructive pulmonary disease, liver cirrhosis, and arterial embolism, and thrombosis. Healthcare utilization was tracked by recording visits to the intensive care unit in the year preceding the observation date and visits to outpatient clinics, emergency departments, and hospital wards within 30 days prior to the observation date. Hemodialysis was defined as current hemodialysis treatment for > 3 months prior to the observation date.

The MPR is an important metric for evaluating medication adherence, calculated by dividing the number of days that a patient has been taking drugs by the number of days in a set observation period, typically 30 days [16]. This ratio, which is a continuous variable, ranges from 0 to 1. It operates under the assumption that the possession of medication implies its consumption, with higher MPR values indicating a better adherence to the prescribed treatment regimen. In addition, any changes in medication were monitored over the preceding 3 months of each observation date. Further, psychotherapy involved formal counseling sessions with a psychiatrist in an outpatient clinic, occurring within 3 months prior to the observation date.

Seasonality was included because shorter daylight and lower temperatures worsen sleep disruption in older adults via photoperiod-driven shifts in melatonin and core temperature [17]. National registries show a 10–20% winter excess in OHCA, attributed to cold-induced sympathetic activation and endothelial stress [18, 19]. While insomnia in older patients has not been directly studied as a modifier, circadian, autonomic, and environmental interactions suggest a plausible association. Seasonal adjustment controls for temporal confounders.

### Identification of OHCA events

OHCA events were identified using the ICD codes and a triage level 1 status in the emergency department [20]. ICD-9 codes 427.4, 427.5, 798, and 798.1 and ICD-10 codes I46, I46.2, I46.8, I46.9, I49.0, I49.01, and R99 were specifically used. The primary outcome measured was the incidence of OHCA events at three time points (the 7th, 30th, and 90th day) after the observation date.

### Model development and internal and Temporal (external) validation

To predict and provide the real-time predictive probabilities of OHCA after each observation date, data spanning from 2011 to 2018 were used for model development. Approximately 80% of the data from each year were allocated as the training set for model construction. Meanwhile, the remaining 20% were used as the internal validation set used in the assessments of performance and reproducibility. In addition, with consideration of the effects of the coronavirus disease 2019 (COVID-19) pandemic, data from 2019 (pre-pandemic) and 2020 (during the pandemic) were selected as the testing sets and used in the temporal validation (external) [21]respectively.

### Statistical analysis

Two independent analysts managed the dataset and conducted statistical analyses using the SAS software (version 9.4, SAS Institute Inc., Cary, NC, the USA) and the R software (version 4.4). The Student’s *t*-test and the chi-square test were utilized to compare continuous and discrete variables, respectively. Multivariate logistic regression analysis with a bidirectional stepwise procedure was performed to examine the association between predictors and OHCA events. Results were reported as adjusted odds ratios and 95% confidence intervals (CIs). A nomogram was constructed to graphically represent each predictor’s impact on the probability of OHCA using a simplified scoring system.

The predictive accuracy of the model was evaluated using the area under the receiver operating characteristic curve (AUROC) across the training, validation, and testing datasets. The Hanley–McNeil test was applied to compare ROC curves (validation vs. testing ROC in 2019, validation vs. testing ROC in 2019, and testing ROC in 2019 vs. testing ROC in 2020). In addition, a calibration plot was used to compare the predicted and observed risks of OHCA.

The overall model performance was assessed using metrics derived from the confusion matrix. The metrics included sensitivity, specificity, accuracy, positive predictive value, negative predictive value, positive likelihood ratio (PLR), negative likelihood ratio (NLR), and diagnostic odds ratio (DOR). Multicollinearity was evaluated with variance-inflation factors (VIFs); all predictors had VIF < 3 (Supplementary Table 2).

## Results

### Participants, training and testing cohorts

**Fig. 1:**
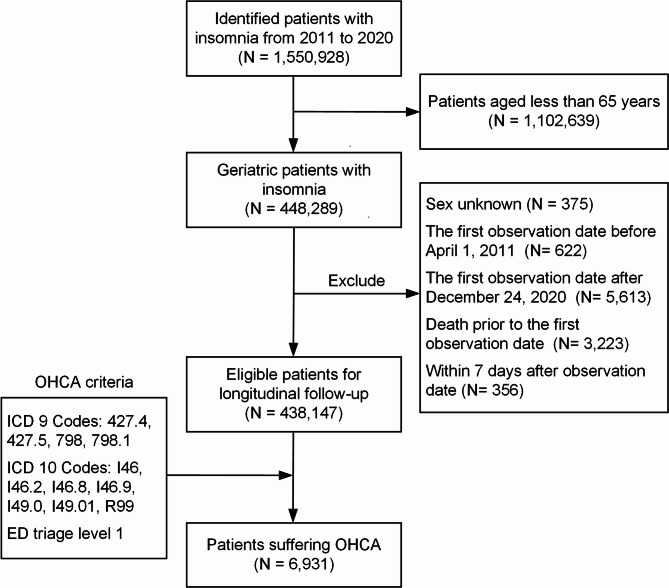
Patient Enrollment Flow: Patients were preliminarily screened, and individuals who met specified exclusion criteria were excluded from the study. The remaining cohort was then subjected to longitudinal long-term follow-up. During the follow-up, incidents of OHCA were identified based on either ICD-9 or ICD-10 diagnostic codes

Figure 1 shows the progression of participant inclusion during the study period from 2011 to 2020. Initially, 1,550,928 patients diagnosed with insomnia were identified. However, only 448,289 (28.9%) of them were a part of the older population. However, due to unknown sex or because the first observation date could not be established for either the initial date or outcome, 10,142 (0.7%) patients were excluded. Consequently, 438,147 eligible patients remained, and they were longitudinally followed-up. Among them, 6,931 (1.6%) experienced OHCA.

The model development cohort comprised 362,678 patients, with an average age of 74.4 years. Approximately 40% of patients were men. The 2019 testing cohort included 40,040 patients, with an average age of 73.7 years. The 2020 testing cohort comprised 35,429 patients, with an average age of 73.5 years. As shown in Supplementary Table 3, there were no significant differences in terms of age or the number of emergency department visits or hospitalizations prior to OHCA between the training and testing cohorts.

### Predictors of OHCA in older patients with insomnia

The current study developed three models to predict the incidence of OHCA at intervals of 7, 30, and 90 days after each observation date. The 7-day OHCA model revealed that older age, male sex, previous use of medical resources (such as intensive care unit admissions, emergency department visits, and hospitalizations), treatment with hemodialysis, the 10 listed pre-existing comorbidities, MPR, medication changes, and psychotherapy were the key predictors of OHCA. The 30-day OHCA model identified the same key predictors as the 7-day model. In addition, the observation month was a significant predictor in this model. Compared with observations in January, other months presented with a lower risk of OHCA occurrence. Similarly, the significant predictors in the 90-day OHCA model were commonly consistent with those in the 30-day model. In addition, November and December were not significantly associated with OHCA incidence compared with January. Supplementary Table 4 shows the details of these associations and their statistical significance.

**Fig. 2:**
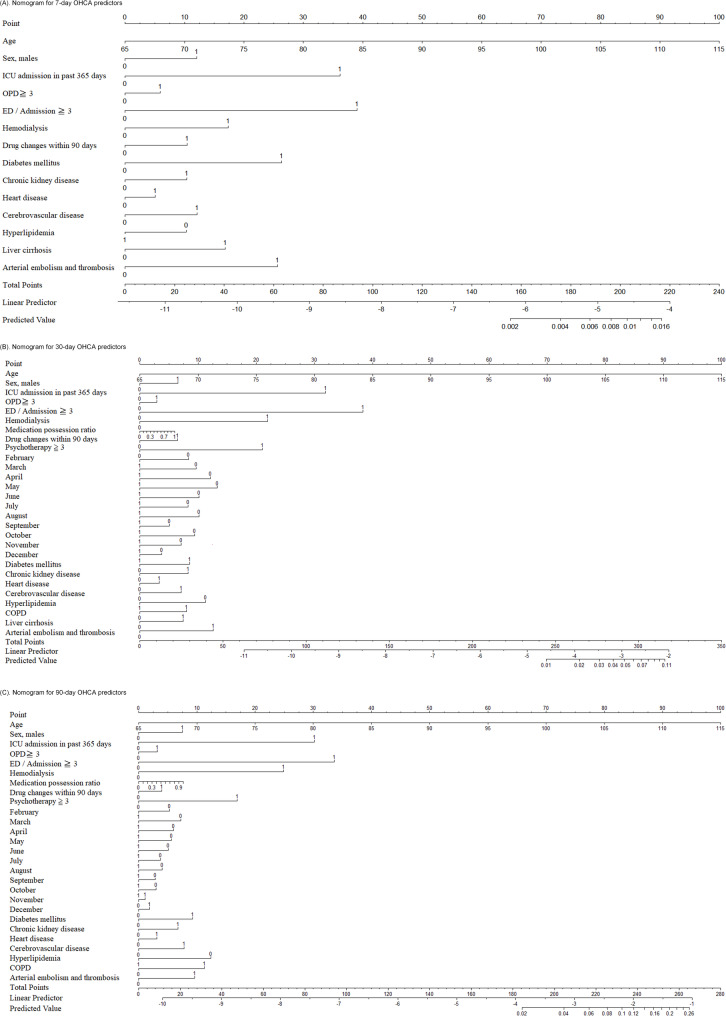
Nomogram for OHCA in Older Patients with Insomnia: **A** 7-day, **B** 30-day; and **C** 90-day. Each individual’s OHCA risk was estimated by plotting on each variable axis. A vertical line was drawn from that value to the top points scale to determine the number of points assigned by that variable value. The points from each variable value were then summed. The sum on the total points scale was located and vertically projected onto the bottom axis, yielding a personalized OHCA risk for older patients with insomnia

Figures 2a, b, and c depict the nomograms for the three prediction models, which depicted the impact of significant predictors on OHCA incidence. Each predictor was represented by a line segment, marked with a scale that reflects the range of values for that variable. Points ranging from 0 to 100 were assigned to each predictor based on its value, and these points were summed up to determine the total points, which ranged from 0 to 240. By drawing a vertical line from the total points down to the baseline, the corresponding predictive probability of OHCA after 7 days (Fig. 2a), 30 days (Fig. 2b), and 90 days (Fig. 2c) after the observation date could be determined.

### Prediction performance of the proposed models

**Fig. 3:**
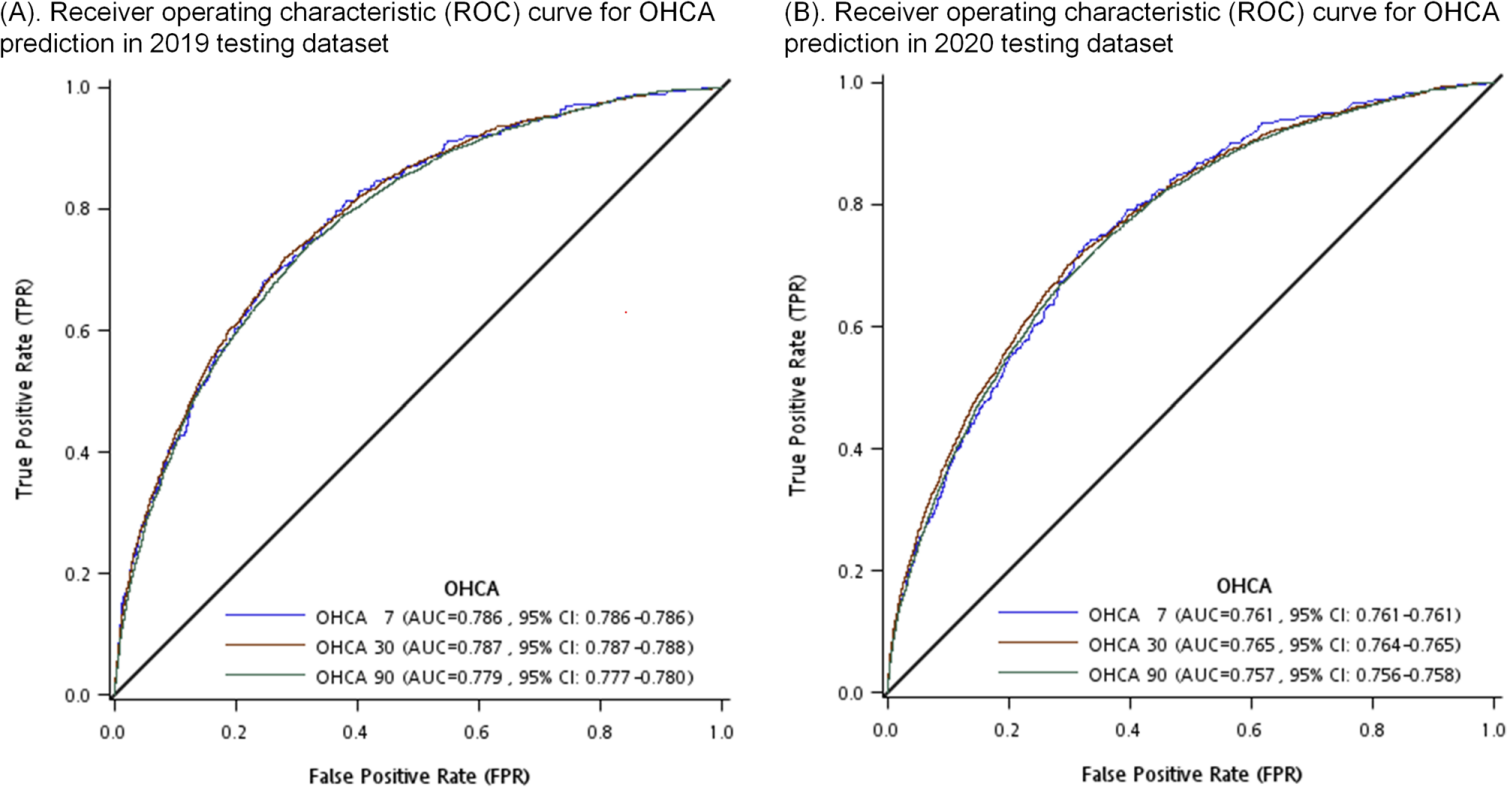
Receiver Operating Characteristic (ROC) Curve for OHCA Prediction: **A** 2019 testing dataset, **B** 2020 testing dataset. In each panel, the ROC curves for three distinct time points—7 days (blue line), 30 days (brown line), and 90 days (green line)—were displayed, along with the area under the curve (AUC) and the corresponding 95% confidence intervals

In the training dataset, the AUROC values ranged from 0.749 to 0.762. In the validation dataset, the AUROC values for the 7-, 30-, and 90-day models were 0.768, 0.768, and 0.748, respectively. For the 2019 testing dataset (Fig. 3a), the AUROC values for the 7-, 30-, and 90-day OHCA models were 0.786 (95% CI: 0.786–0.786), 0.787 (95% CI: 0.787–0.788), and 0.779 (95% CI: 0.777–0.780), respectively. In the 2020 testing dataset (Fig. 3b), the respective AUROC values for the 7-, 30-, and 90-day models were 0.761 (95% CI: 0.761–0.761), 0.765 (95% CI: 0.764–0.765), and 0.757 (95% CI: 0.756–0.758), respectively.

Table 1 presents the overall prediction performance of the model derived from the confusion matrix, including the validation dataset and two testing datasets. In particular, for predicting short-term OHCA, the 7-day OHCA model presented with the following performance metrics in the 2019 testing dataset: sensitivity, 0.781; specificity, 0.754; accuracy, 0.754; PLR, 2.78; NLR, 0.42; and DOR, 6.58. In the 2020 testing dataset, the sensitivity, specificity, accuracy, PLR, NLR, and DOR of the 7-day model were 0.731, 0.677, 0.677, 2.27, 0.40, and 5.71, respectively.

**Table 1:** Performance of models in validation and testing dataset

	Model: 7-day OHCA			Model: 30-day OHCA			Model: 90-day OHCA		
	Validation	Testing (2019)	Testing (2020)	Validation	Testing (2019)	Testing (2020)	Validation	Testing (2019)	Testing (2020)
Sensitivity	0.710	0.781	0.731	0.726	0.721	0.704	0.742	0.741	0.669
Specificity	0.685	0.754	0.677	0.690	0.718	0.698	0.658	0.681	0.715
Accuracy	0.685	0.754	0.677	0.690	0.718	0.698	0.658	0.681	0.715
Positive predictive value	0.000	0.000	0.000	0.001	0.001	0.001	0.002	0.002	0.002
Negative predictive value	0.999	0.999	0.999	0.999	0.999	0.999	0.999	0.999	0.999
Positive likelihood ratio	2.258	2.775	2.265	2.342	2.561	2.338	2.173	2.327	2.353
Negative likelihood ratio	0.421	0.421	0.396	0.396	0.388	0.422	0.391	0.379	0.462
Diagnostic odds ratio	5.351	6.580	5.712	5.903	6.593	5.528	5.557	6.127	5.089

Further, for predicting long-term OHCA, the sensitivity, specificity, accuracy, PLR, NLR, and DOR of the 90-day OHCA model in the 2019 testing dataset were 0.741, 0.681, 0.681, 2.33, 0.38, and 6.13, respectively. In the 2020 testing dataset, the sensitivity, specificity, accuracy, PLR, NLR, and DOR of the model registered were 0.669, 0.715, 0.715, 2.35, 0.46, and 5.09, respectively.

### Calibration

**Fig. 4:**
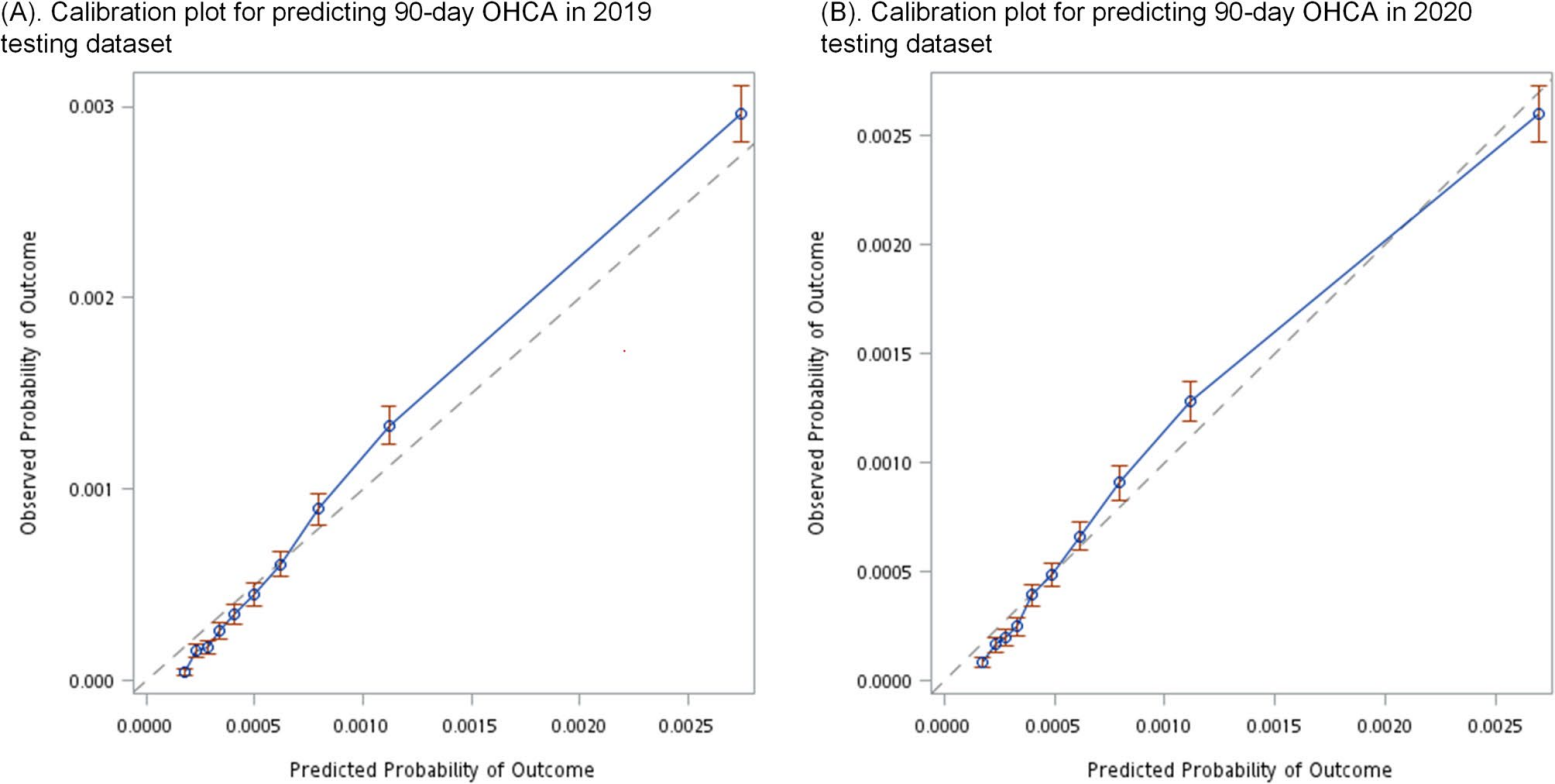
Calibration Plots for Predicting 90-day OHCA: **A** 2019 testing dataset, **B** 2020 testing dataset. The dotted line at 45 degrees indicates perfect calibration, where predicted and observed probabilities are equal. The 10 dots (with red confidence intervals) represent tenths of the population divided based on predicted probability

Figure 4 depicts the calibration plot for predicting 90-day OHCA in the 2019 and 2020 testing datasets. The appendix shows the calibration plots for 7- and 30-day OHCA. The dotted 45-degree line indicated a perfect calibration, where the predicted and observed probabilities were equal. Considering the low rate of OHCA in the population, the predicted and observed probabilities ranged from 0 to 0.003. The 10 dots (with CIs) represented deciles of the population divided based on the predicted probability. In the 2019 dataset, the model’s performance for OHCA prediction was slightly underestimated, as indicated by the blue line commonly observed above the dotted line. Meanwhile, in the 2020 dataset, the predicted and observed probabilities were significantly closer.

### Sensitivity analysis

Cognitive impairment and dementia are well-established contributors to sleep disturbances in older adults. To minimize the risk of misclassification bias, we accounted for pre-existing comorbidities, including dementia, mild cognitive impairment, and depressive disorders. In the multivariable model, none of these three conditions were significantly associated with 7-day, 30-day, or 90-day OHCA (Supplementary Table 5).

## Discussion

### Brief summary and study strengths

In this study, older patients with insomnia presented with various risks of OHCA and associated predictors across different time points. Older age was the primary predictor of OHCA. The additional predictors included recent admission or visit to medical institutions, pre-existing comorbidities, the MPR for insomnia medications, recent psychotherapy, and months potentially linked to variations in ambient temperature. The current study had several strengths. That is, it was a large-scale study, and it had an 8-year longitudinal follow-up using a panel data structure. This design not only identifies the predictors of OHCA but also directly quantifies and calculates both the short and long-term risks of OHCA. Further, our approach in the external validation significantly enhances the generalizability and credibility of our findings. These insights can provide emergency physicians and psychiatrists with essential information on clinical practice, particularly in managing the potential risks of OHCA in older patients with insomnia.

### Prediction of short-term OHCA in older patients with insomnia

The primary predictors of short-term OHCA in older patients with insomnia were primarily attributed to inherent health conditions and therapeutic interventions. Age was a key variable in the nomogram, with each incremental 5-year increase elevating the patient’s score by 10 points. If the cumulative score was > 150, the probability of experiencing OHCA within 7 days increased to 0.2%, thereby significantly exceeding the annual incidence rate of approximately 35–70 cases per 100,000 individuals. Further, frequent utilization of healthcare resources, such as emergency, outpatient, and inpatient services, may indicate the emergence or worsening of pre-existing disease conditions. In addition to the prevalent comorbidities among older individuals, attending more than three psychotherapy (counseling) sessions within 90 days may imply a worsening or underlying insomnia. Changes in pharmacological treatments increased the risk of short-term OHCA. In contrast, environmental factors had a minimal impact on OHCA incidence. This is likely attributed to the need for prolonged exposure or a cumulative dose effect [22]. Notably, ambient temperature fluctuations did not significantly affect predicting short-term OHCA in older patients with insomnia.

### Prediction of long-term OHCA in older patients with insomnia

Combined with the predictors of short-term OHCA, the MPR was specifically essential in predicting long-term OHCA occurrence. Every 1% increase in MPR was correlated with a 1.26-fold increase in the odds of experiencing OHCA within 90 days. Hence, older patients with insomnia might benefit from therapeutic approaches other than pharmacotherapy. In addition to medication adherence, ambient temperature also played a role in predicting long-term OHCA occurrences. Using the coldest month as a reference, our study showed that the odds of OHCA in months other than January were lower, thereby emphasizing a potential elevated risk of sudden death during the winter months in older patients with insomnia. This risk further increased when MPR values were added. In addition, our nomogram showed that a total score of 180 corresponded to an OHCA probability of 0.5% in the short-term model (Fig. 2a), which increased to 2% in the long-term model (Fig. 2c). Therefore, there is an increasing cumulative probability of OHCA over time. While the model demonstrates adequate sensitivity and specificity, its low PPV suggests limited clinical utility in identifying individual high-risk patients.

### Effects of COVID-19 on the predictors and prediction performance

Previous studies showed that in response to the COVID-19 pandemic, various countries and healthcare institutions implemented isolation and protective measures. These policies indirectly affected public lifestyles, causing changes in both the incidence and outcome determinants of OHCA [23–25]. This study used the 2019 and 2020 cohorts as two independent testing datasets. This approach facilitated the evaluation of the model’s efficacy under different conditions—both prior to and during the pandemic. The 2019 dataset evaluates the model’s performance in a non-pandemic environment. Meanwhile, the 2020 dataset offers insights into its applicability during the pandemic, thereby allowing a comparative analysis of the effect of global health crisis on OHCA event prediction in older patients with insomnia. Results showed no significant difference between the prediction performance validation and testing datasets (*p* = 0.721 in the Hanley–McNeil test). One explanation for the observed patterns was that the older population seldom leaves their houses, with some even remaining completely housebound. The effects of quarantine and isolation policies during the pandemic among older individuals were relatively minor compared with those experienced by younger individuals. Hence, no significant differences were observed between the two testing datasets in older patients with insomnia. However, whether the anxiety induced by the pandemic indirectly influenced the severity of insomnia in this group remains an open question that should be further investigated.

### Iatrogenic effects of sleeping drugs

Our models demonstrate that a higher MPR for hypnotics—a proxy for sustained, high-intensity exposure—is associated with a stepwise increase in 90-day OHCA risk (adjusted OR 1.26). Pharmacologically, benzodiazepine receptor agonists and sedating antipsychotics are known to prolong the QT interval, blunt adrenergic arousal, and depress ventilatory drive; long-term receptor downregulation may further heighten vulnerability during acute stressors [26, 27]. Additionally, polypharmacy with antihypertensives or antiarrhythmics could potentiate these effects, suggesting that strict hypnotic adherence itself—rather than insomnia severity alone—may partially mediate the observed association.

### Clinical implications

Embedding the findings in hospital information systems could automate flagging at prescription renewal, prompting medication review, cardiology referral, and sleep-behavior counselling. For emergency medical service, season-stratified risk curves support winter-focused education and resource allocation. Targeted interventions for this high-risk group may reduce the incidence of OHCA. However, because Taiwan’s health insurance structure ensures nearly complete data capture, replication in countries with different insurance models, prescribing cultures, and geriatric care pathways is required before broad implementation. Prospective validation in diverse populations would be necessary to establish the model’s transportability and allow recalibration where appropriate.

### Study limitations

The current study had some limitations. First, sleep deprivation and delirium were significantly connected. That is, they exhibited common clinical features, risk factors, and neurochemical irregularities [28]. Hence, in this study, older individuals initially diagnosed with insomnia might have been experiencing delirium, potentially leading to selection bias. Second, not all older patients with insomnia personally had clinic visits. Due to disability, some individuals might have prescriptions collected by their family members. This prevented psychiatrists from directly assessing the patient’s condition. However, this study hypothesized that these patients are stable and at lower risk of OHCA. This finding was supported by the fact that undergoing more than three psychotherapy sessions within 3 months—a predictor of OHCA due to potential instability or worsening condition—could serve as a cross-validation. Third, although the associations between poor medication adherence and 30-day OHCA remained robust across sensitivity analyses, unmeasured psychiatric comorbidity and obstructive sleep apnea could not be fully excluded and may partly account for the magnitude of risk reported. Fourth, vascular cognitive impairment and other forms of cognitive decline were not included in this study because these conditions are often misclassified in administrative claims data. Consequently, we were unable to adjust for them; residual confounding by unrecognized cognitive decline may therefore attenuate or amplify the observed associations between insomnia, medication adherence, and short-term OHCA risk. Fifth, residual confounding in either direction of insomnia-related counseling may be possible. Because psychotherapy utilization in the database likely reflects refractory insomnia and psychological distress, our analysis cannot distinguish the therapeutic benefit of counseling from the risk conferred by the severity it signifies. Sixth, although we performed additional multivariable logistic regression analyses to examine potential changes in the predictors’ impact on OHCA risk—resulting in 24 year-specific models—we did not conduct a trend analysis of their significance. Finally, our cohort covered > 95% of the whole population. However, not all patients with OHCAs were brought to hospitals, and not all patients with OHCA were transported to hospitals. Thus, the reported incidence of OHCA events was underestimated.

## Conclusions

In addition to age, sex, and pre-existing comorbidities, MPR, recent changes in insomnia medications, and frequent psychotherapy counseling can be predictive factors of 7- or 90-day OHCA in older patients with insomnia.

## Supplementary Information

Below is the link to the electronic supplementary material.

Supplementary Material 1

## Data Availability

No datasets were generated or analysed during the current study.
