# Supplementary material for: Prediction of out-of-hospital cardiac arrest in older patients with insomnia: a longitudinal population study
Source: BMC Geriatr. 2025 Aug 7;25:600. doi: 10.1186/s12877-025-06285-x (PMC12329982; doi:10.1186/s12877-025-06285-x)
Supplement: Supplementary file 1 — Supplementary Material 1 [file 12877_2025_6285_MOESM1_ESM.docx]

| **Table S1. Drugs for insomnia in geriatric patients** | | |
| --- | --- | --- |
| **Classification** | **Drugs** | **ATC Code** |
| Benzodiazepines | Alprazolam | N05BA12 |
| Benzodiazepines | Bromazepam | N05BA08 |
| Benzodiazepines | Brotizolam | N05CD09 |
| Benzodiazepines | Diazepam | N05BA01 |
| Benzodiazepines | Estazolam | N05CD04 |
| Benzodiazepines | Flunitrazepam | N05CD03 |
| Benzodiazepines | Lorazepam | N05BA06 |
| Benzodiazepines | Oxazolam | N05BA91 |
| Sedatives | Buspirone Hcl | N05BE01 |
| Sedatives | Zolpidem Hemitartrate | N05CF02 |
| Sedatives | Zolpidem Tartrate | N05CF02 |
| Sedatives | Zopiclone | N05CF01 |
| Antipsychotics | Amisulpride | N05AL05 |
| Antipsychotics | Aripiprazole | N05AX12 |
| Antipsychotics | Aripiprazole Monohydrate | N05AX12 |
| Antipsychotics | Brexpiprazole | N05AX16 |
| Antipsychotics | Chlorpromazine (Hcl) | N05AA01 |
| Antipsychotics | Chlorpromazine | N05AA01 |
| Antipsychotics | Clozapine | N05AH02 |
| Antipsychotics | Haloperidol | N05AD01 |
| Antipsychotics | Haloperidol (Decanoate) | N05AD01 |
| Antipsychotics | Lurasidone Hcl | N05AE05 |
| Antipsychotics | Olanzapine | N05AH03 |
| Antipsychotics | Olanzapine Micronized | N05AH03 |
| Antipsychotics | Paliperidone | N05AX13 |
| Antipsychotics | Prochlorperazine ((Di)Maleate) | N05AB04 |
| Antipsychotics | Prochlorperazine (Maleate) | N05AB04 |
| Antipsychotics | Prochlorperazine Dimethanesulfonate | N05AB04 |
| Antipsychotics | Risperidone | N05AX08 |
| Antipsychotics | Sulpiride | N05AL01 |
| Antipsychotics | Ziprasidone Hydrochloride | N05AE04 |
| Antidepressants | Agomelatine | N06AX22 |
| Antidepressants | Bupropion Hydrochloride | N06AX12 |
| Antidepressants | Doxepin Hcl | N06AA12 |
| Antidepressants | Duloxetine( Hydrochloride) | N06AX21 |
| Antidepressants | Escitalopram (As Oxalate) | N06AB10 |
| Antidepressants | Fluoxetine (Hcl) | N06AB03 |
| Antidepressants | Flupentixol 2hcl／Melitracen Hcl | N06CA02 |
| Antidepressants | Imipramine Hcl | N06AA02 |
| Antidepressants | Mirtazapine | N06AX11 |
| Antidepressants | Paroxetine Hydrochloride | N06AB05 |
| Antidepressants | Sertraline(As Hydrochloride) | N06AB06 |
| Antidepressants | Sertraline Hydrochloride | N06AB06 |
| Antidepressants | Trazodone Hydrochloride | N06AX05 |
| Antidepressants | Venlafaxine (Hcl) | N06AX16 |
| Antidepressants | Vortioxetine Hydrobromide | N06AX26 |
| Miscellaneous | Atomoxetine (Hydrochloride) | N06BA09 |
| Miscellaneous | Dihydroergotoxine Methanesulfonate | C04AE01 |
| Miscellaneous | Dihydroergotoxine | C04AE01 |
| Miscellaneous | Methylphenidate Hcl | N06BA04 |
| Miscellaneous | Modafinil | N06BA07 |
| Miscellaneous | Piracetam | N06BX03 |
| Miscellaneous | Pitolisant Hydrochloride | N07XX11 |

| **Table S2. The multicollinearity analysis in three models** | | | | | |
| --- | --- | --- | --- | --- | --- |
| **Model: 7-day OHCA** | | **Model: 30-day OHCA** | | **Model: 90-day OHCA** | |
| **Variables** | **VIF** | **Variables** | **VIF** | **Variables** | **VIF** |
| Age | 1.015 | Age | 1.018 | Age | 1.018 |
| Sex, males | 1.011 | Sex, males | 1.023 | Sex, males | 1.023 |
| ICU admission | 1.027 | ICU admission | 1.028 | ICU admission | 1.028 |
| OPD visits ≥ 3 | 1.027 | OPD visits ≥ 3 | 1.031 | OPD visits ≥ 3 | 1.030 |
| Admission or ED visit ≥ 3 | 1.021 | Admission or ED visit ≥ 3 | 1.022 | Admission or ED visit ≥ 3 | 1.022 |
| Hemodialysis | 1.017 | Hemodialysis | 1.176 | Hemodialysis | 1.176 |
| Drug changes within 90 days | 1.007 | Medication possession ratio | 1.034 | Medication possession ratio | 1.034 |
| Psychotherapy ≥ 3 | 1.002 | Drug changes within 90 days | 1.032 | Drug changes within 90 days | 1.032 |
| Diabetes Mellitus | 1.095 | Psychotherapy ≥ 3 | 1.003 | Psychotherapy ≥ 3 | 1.003 |
| Heart disease | 1.036 | February | 1.809 | February | 1.809 |
| Cerebrovascular disease | 1.018 | March | 1.906 | March | 1.906 |
| Hyperlipidemia | 1.107 | April | 1.953 | April | 1.953 |
| Cirrhosis | 1.004 | May | 2.035 | May | 2.035 |
| Arterial embolism and thrombosis | 1.007 | June | 2.037 | June | 2.037 |
|  |  | July | 2.077 | July | 2.077 |
|  |  | August | 2.084 | August | 2.084 |
|  |  | September | 2.063 | September | 2.063 |
|  |  | October | 2.117 | October | 2.117 |
|  |  | November | 2.112 | November | 2.112 |
|  |  | December | 2.167 | December | 2.167 |
|  |  | Diabetes Mellitus | 1.101 | Diabetes Mellitus | 1.099 |
|  |  | Chronic kidney disease | 1.186 | Chronic kidney disease | 1.186 |
|  |  | Heart disease | 1.042 | Heart disease | 1.042 |
|  |  | Cerebrovascular disease | 1.019 | Cerebrovascular disease | 1.019 |
|  |  | Hyperlipidemia | 1.109 | Hyperlipidemia | 1.108 |
|  |  | Chronic obstructive pulmonary disease | 1.016 | Chronic obstructive pulmonary disease | 1.016 |
|  |  | Cirrhosis | 1.004 | Arterial embolism and thrombosis | 1.008 |
|  |  | Arterial embolism and thrombosis | 1.008 |  |  |
| ED: emergency department; OPD: outpatient department | | | | | |

| **Table S3. Comparison of characteristics in training dataset and testing dataset** | | | | | | |
| --- | --- | --- | --- | --- | --- | --- |
| **Predictors** | **Training (N=362,678)** | **Testing (2019) N=(40,040)** | **Testing (2020) N=(35,429)** | ***p^α^*** | ***p^β^*** | ***p*^γ^** |
| **Demographics and health conditions** |  |  |  |  |  |  |
| Age | 74.35 ± 6.47 | 73.72 ± 6.67 | 73.53 ± 6.59 | 0.087 | 0.068 | 0.312 |
| Sex, males | 146338 (40.35) | 16720 (41.76) | 15026 (42.41) | <0.001 | <0.001 | 0.070 |
| ICU admission in past 365 days | 14662 (4.04) | 1752 (4.38) | 1585 (4.47) | 0.001 | <0.001 | 0.513 |
| Outpatient clinics more than 3 times in past 30 days | 159958 (44.10) | 16364 (40.87) | 14201 (40.08) | <0.001 | <0.001 | 0.028 |
| ED and/or ward hospitalization more than 3 times in past 30 days | 7706 (2.12) | 901 (2.25) | 717 (2.02) | 0.099 | 0.207 | 0.032 |
| Hemodialysis | 4963 (1.37) | 636 (1.59) | 617 (1.74) | 0.000 | <0.001 | 0.100 |
| **Pre-existing comorbidities** |  |  |  |  |  |  |
| Diabetes mellitus | 76501 (21.09) | 8401 (20.98) | 7743 (21.85) | 0.603 | 0.001 | 0.004 |
| Hypertension | 169599 (46.76) | 16773 (41.89) | 15438 (43.57) | <0.001 | <0.001 | <0.001 |
| Chronic kidney disease | 18137 (5.00) | 2403 (6.00) | 2384 (6.73) | <0.001 | <0.001 | <0.001 |
| Peptic ulcer disease | 49016 (13.52) | 4384 (10.95) | 3953 (11.16) | <0.001 | <0.001 | 0.362 |
| Heart disease | 77526 (21.38) | 7339 (18.33) | 6867 (19.38) | <0.001 | <0.001 | 0.000 |
| Cerebrovascular disease | 27675 (7.63) | 2405 (6.01) | 2299 (6.49) | <0.001 | <0.001 | 0.006 |
| Hyperlipidemia | 74353 (20.5) | 7804 (19.49) | 7140 (20.15) | <0.001 | 0.121 | 0.023 |
| Chronic obstructive pulmonary disease | 8841 (2.44) | 726 (1.81) | 676 (1.91) | <0.001 | <0.001 | 0.336 |
| Liver cirrhosis | 5072 (1.4) | 500 (1.25) | 468 (1.32) | 0.015 | 0.234 | 0.379 |
| Arterial embolism and thrombosis | 2296 (0.63) | 197 (0.49) | 158 (0.45) | 0.001 | <0.001 | 0.356 |
| **Insomnia therapy** |  |  |  |  |  |  |
| Medication possession ratio | 0.43 ± 0.33 | 0.47 ± 0.33 | 0.53 ± 0.33 | <0.001 | <0.001 | <0.001 |
| Drug changes within 90 days | 37255 (10.28) | 6524 (16.3) | 6713 (18.95) | <0.001 | <0.001 | <0.001 |
| Psychotherapy | 28464 (7.85) | 5118 (12.78) | 5115 (14.44) | <0.001 | <0.001 | <0.001 |
| Psychotherapy more than 3 times in past 30 days | 3172 (0.87) | 820 (2.05) | 966 (2.73) | <0.001 | <0.001 | <0.001 |
| **Observation month** |  |  |  | <0.001 | <0.001 | <0.001 |
| January | 25351 (6.99) | 3423 (8.55) | 3420 (9.65) |  |  |  |
| February | 23646 (6.52) | 3090 (7.72) | 3061 (8.64) |  |  |  |
| March | 26671 (7.35) | 3277 (8.18) | 3109 (8.78) |  |  |  |
| April | 41125 (11.34) | 3495 (8.73) | 3542 (10) |  |  |  |
| May | 41207 (11.36) | 2995 (7.48) | 3175 (8.96) |  |  |  |
| June | 38323 (10.57) | 3425 (8.55) | 3408 (9.62) |  |  |  |
| July | 32432 (8.94) | 3377 (8.43) | 3227 (9.11) |  |  |  |
| August | 28467 (7.85) | 3132 (7.82) | 2996 (8.46) |  |  |  |
| September | 25335 (6.99) | 3596 (8.98) | 2599 (7.34) |  |  |  |
| October | 25670 (7.08) | 3489 (8.71) | 2381 (6.72) |  |  |  |
| November | 26506 (7.31) | 3187 (7.96) | 2430 (6.86) |  |  |  |
| December | 27945 (7.71) | 3554 (8.88) | 2081 (5.87) |  |  |  |
| ED: emergency department; ICU: intensive care unit  *p*^α^: Comparison between training dataset and testing dataset (in year 2019)  *p*^β^: Comparison between training dataset and testing dataset (in year 2020)  *p*^γ^: Comparison between testing dataset (in year 2019) and testing dataset (in year 2020) | | | | | | |

| **Table S4. Predictive regression models for the incidence of out-of-hospital cardiac arrest at 7-, 30-, and 90-days following observation date** | | | | | | | | | | | |
| --- | --- | --- | --- | --- | --- | --- | --- | --- | --- | --- | --- |
| **Model type** | **Model: 7-day OHCA** | | |  | **Model: 30-day OHCA** | | |  | **Model: 90-day OHCA** | | |
|  | aOR | 95% CI | p |  | aOR | 95% CI | p |  | aOR | 95% CI | p |
| **Demographics and health conditions** |  |  |  |  |  |  |  |  |  |  |  |
| Age | 1.07 | 1.06-1.08 | <0.001 |  | 1.07 | 1.07-1.08 | <0.001 |  | 1.07 | 1.07-1.08 | <0.001 |
| Sex, males | 1.62 | 1.50-1.76 | <0.001 |  | 1.39 | 1.34-1.44 | <0.001 |  | 1.41 | 1.38-1.44 | <0.001 |
| ICU admission in past 365 days | 3.17 | 2.83-3.55 | <0.001 |  | 3.22 | 3.05-3.39 | <0.001 |  | 2.95 | 2.85-3.04 | <0.001 |
| Outpatient clinics more than 3 times in past 30 days | 1.19 | 1.10-1.29 | 0.001 |  | 1.10 | 1.06-1.14 | 0.001 |  | 1.10 | 1.07-1.12 | <0.001 |
| ED and/or ward hospitalization more than 3 times in past 30 days | 4.39 | 3.91-4.92 | <0.001 |  | 4.37 | 4.13-4.62 | <0.001 |  | 3.61 | 3.49-3.73 | <0.001 |
| Hemodialysis | 2.82 | 2.36-3.37 | <0.001 |  | 2.66 | 2.42-2.93 | <0.001 |  | 2.81 | 2.66-2.96 | <0.001 |
| **Pre-existing comorbidities** |  |  |  |  |  |  |  |  |  |  |  |
| Diabetes mellitus | 1.53 | 1.40-1.67 | <0.001 |  | 1.44 | 1.39-1.51 | <0.001 |  | 1.45 | 1.41-1.49 | <0.001 |
| Hypertension |  |  |  |  |  |  |  |  |  |  |  |
| Chronic kidney disease |  |  |  |  | 1.29 | 1.20-1.37 | <0.001 |  | 1.29 | 1.24-1.34 | <0.001 |
| Peptic ulcer disease |  |  |  |  |  |  |  |  |  |  |  |
| Heart disease | 1.15 | 1.05-1.67 | <0.001 |  | 1.12 | 1.07-1.17 | <0.001 |  | 1.13 | 1.10-1.16 | <0.001 |
| Cerebrovascular disease | 1.45 | 1.31-1.62 | <0.001 |  | 1.38 | 1.31-1.45 | <0.001 |  | 1.38 | 1.34-1.42 | <0.001 |
| Hyperlipidemia | 0.68 | 0.61-0.76 | <0.001 |  | 0.63 | 0.60-0.67 | <0.001 |  | 0.63 | 0.61-0.65 | <0.001 |
| Chronic obstructive pulmonary disease |  |  |  |  | 1.50 | 1.39-1.62 | <0.001 |  | 1.59 | 1.53-1.66 | <0.001 |
| Liver cirrhosis | 1.25 | 0.94-1.67 | 0.131 |  | 1.00 | 0.86-1.17 | 0.957 |  |  |  |  |
| Arterial embolism and thrombosis | 1.67 | 1.27-2.19 | <0.001 |  | 1.26 | 1.09-1.46 | 0.002 |  | 1.24 | 1.13-1.35 | <0.001 |
| **Insomnia therapy** |  |  |  |  |  |  |  |  |  |  |  |
| Medication possession ratio |  |  |  |  | 1.18 | 1.12-1.25 | <0.001 |  | 1.26 | 1.22-1.30 | <0.001 |
| Drug changes within 90 days | 1.50 | 1.26-1.79 | <0.001 |  | 1.24 | 1.13-1.36 | <0.001 |  | 1.14 | 1.09-1.21 | <0.001 |
| Psychotherapy more than 3 times in past 30 days | 1.69 | 1.04-2.77 | 0.036 |  | 1.79 | 1.42-2.25 | <0.001 |  | 1.99 | 1.76-2.26 | <0.001 |
| **Observation month** |  |  |  |  |  |  |  |  |  |  |  |
| January |  |  |  |  | Ref | - | - |  | Ref | - | - |
| February |  |  |  |  | 0.82 | 0.74-0.89 | <0.001 |  | 0.87 | 0.82-0.92 | <0.001 |
| March |  |  |  |  | 0.79 | 0.73-0.87 | <0.001 |  | 0.86 | 0.81-0.91 | <0.001 |
| April |  |  |  |  | 0.71 | 0.64-0.77 | <0.001 |  | 0.87 | 0.83-0.92 | <0.001 |
| May |  |  |  |  | 0.76 | 0.69-0.83 | <0.001 |  | 0.87 | 0.83-0.92 | <0.001 |
| June |  |  |  |  | 0.78 | 0.72-0.86 | <0.001 |  | 0.85 | 0.80-0.89 | <0.001 |
| July |  |  |  |  | 0.75 | 0.68-0.82 | <0.001 |  | 0.88 | 0.83-0.92 | <0.001 |
| August |  |  |  |  | 0.72 | 0.66-0.79 | <0.001 |  | 0.89 | 0.85-0.94 | <0.001 |
| September |  |  |  |  | 0.85 | 0.78-0.93 | <0.001 |  | 0.92 | 0.87-0.97 | <0.001 |
| October |  |  |  |  | 0.78 | 0.72-0.86 | <0.001 |  | 0.96 | 0.91-1.01 | 0.105 |
| November |  |  |  |  | 0.81 | 0.74-0.88 | <0.001 |  | 1.07 | 1.01-1.12 | 0.013 |
| December |  |  |  |  | 0.98 | 0.91-1.07 | 0.749 |  | 1.08 | 1.03-1.14 | 0.002 |
| aOR: adjusted odds ratio; CI: confidence interval; ED: emergency department; ICU: intensive care unit; OHCA: out-of-hospital cardiac arrest | | | | | | | | | | | |

| **Table S5. Sensitivity analysis: predictive regression models for the incidence of out-of-hospital cardiac arrest at 7-, 30-, and 90-days following observation date (including dementia, cognitive impairment, and depressive disorders)** | | | | | | | | | | | |
| --- | --- | --- | --- | --- | --- | --- | --- | --- | --- | --- | --- |
| **Model type** | **Model: 7-day OHCA** | | |  | **Model: 30-day OHCA** | | |  | **Model: 90-day OHCA** | | |
|  | aOR | 95% CI | p |  | aOR | 95% CI | p |  | aOR | 95% CI | p |
| **Demographics and health conditions** |  |  |  |  |  |  |  |  |  |  |  |
| Age | 1.07 | 1.06-1.08 | <0.001 |  | 1.06 | 1.05-1.08 | <0.001 |  | 1.07 | 1.06-1.08 | <0.001 |
| Sex, males | 1.60 | 1.52-1.74 | <0.001 |  | 1.41 | 1.29-1.45 | <0.001 |  | 1.45 | 1.33-1.62 | <0.001 |
| ICU admission in past 365 days | 3.02 | 2.32-3.52 | <0.001 |  | 3.21 | 3.02-3.33 | <0.001 |  | 2.67 | 2.05-3.34 | <0.001 |
| Outpatient clinics more than 3 times in past 30 days | 1.08 | 1.00-1.17 | 0.021 |  | 1.09 | 1.04-1.15 | 0.002 |  | 1.11 | 1.09-1.14 | <0.001 |
| ED and/or ward hospitalization more than 3 times in past 30 days | 4.65 | 3.98-5.21 | <0.001 |  | 4.33 | 4.11-4.61 | <0.001 |  | 3.68 | 3.43-3.79 | <0.001 |
| Hemodialysis | 2.45 | 2.09-3.12 | <0.001 |  | 2.64 | 2.32-2.87 | <0.001 |  | 2.78 | 2.60-2.99 | <0.001 |
| **Pre-existing comorbidities** |  |  |  |  |  |  |  |  |  |  |  |
| Diabetes mellitus | 1.38 | 1.17-1.85 | <0.001 |  | 1.42 | 1.37-1.50 | <0.001 |  | 1.44 | 1.36-1.53 | <0.001 |
| Chronic kidney disease |  |  |  |  | 1.22 | 1.10-1.38 | <0.001 |  | 1.27 | 1.23-1.39 | <0.001 |
| Heart disease | 1.09 | 1.03-1.41 | <0.001 |  | 1.10 | 1.05-1.19 | <0.001 |  | 1.15 | 1.09-1.23 | <0.001 |
| Cerebrovascular disease | 1.38 | 1.22-1.57 | <0.001 |  | 1.37 | 1.30-1.44 | <0.001 |  | 1.37 | 1.30-1.40 | <0.001 |
| Hyperlipidemia | 0.63 | 0.55-0.78 | <0.001 |  | 0.55 | 0.42-0.69 | <0.001 |  | 0.54 | 0.43-0.69 | <0.001 |
| Chronic obstructive pulmonary disease |  |  |  |  | 1.42 | 1.22-1.57 | <0.001 |  | 1.55 | 1.51-1.65 | <0.001 |
| Liver cirrhosis | 1.21 | 0.96-1.537 | 0.208 |  | 1.01 | 0.87-1.19 | 0.904 |  |  |  |  |
| Arterial embolism and thrombosis | 1.62 | 1.26-2.18 | <0.001 |  | 1.25 | 1.11-1.45 | 0.003 |  | 1.21 | 1.11-1.35 | <0.001 |
| Dementia* | 1.35 | 0.87-1.56 | 0.263 |  | 1.22 | 0.91-1.60 | 0.298 |  | 1.32 | 0.99-1.49 | 0.051 |
| Mild cognitive impairment* | 1.21 | 0.79-1.43 | 0.563 |  | 1.16 | 0.82-1.30 | 0.432 |  | 1.21 | 0.92-1.28 | 0.075 |
| Depressive disorders* | 1.02 | 0.67-1.22 | 0.733 |  | 1.08 | 0.72-1.18 | 0.764 |  | 0.98 | 0.78-1.29 | 0.234 |
| **Insomnia therapy** |  |  |  |  |  |  |  |  |  |  |  |
| Medication possession ratio |  |  |  |  | 1.09 | 1.06-1.21 | <0.001 |  | 1.21 | 1.13-1.43 | <0.001 |
| Drug changes within 90 days | 1.34 | 1.27-1.43 | <0.001 |  | 1.29 | 1.09-1.43 | <0.001 |  | 1.19 | 1.06-1.32 | <0.001 |
| Psychotherapy more than 3 times in past 30 days | 1.63 | 1.02-1.98 | 0.006 |  | 1.67 | 1.40-2.05 | <0.001 |  | 2.32 | 1.36-2.87 | <0.001 |
| aOR: adjusted odds ratio; CI: confidence interval; ED: emergency department; ICU: intensive care unit; OHCA: out-of-hospital cardiac arrest | | | | | | | | | | | |

*identified by the ICD codes
